# Exploring the depths of on‐water training in highly‐trained rowing athletes

**DOI:** 10.1002/ejsc.12069

**Published:** 2024-03-18

**Authors:** Sophie P. Watts, Martyn J. Binnie, Paul S.R. Goods, Jamie Hewlett, Peter Peeling

**Affiliations:** ^1^ School of Human Sciences (Exercise and Sport Science) The University of Western Australia Crawley Western Australia Australia; ^2^ Western Australian Institute of Sport Mount Claremont Western Australia Australia; ^3^ Murdoch Applied Sports Science Laboratory School of Allied Health Murdoch University Perth Western Australia Australia; ^4^ Centre for Healthy Ageing Health Futures Institute Murdoch University Perth Western Australia Australia

**Keywords:** environmental conditions, heart‐rate, stroke‐rate, velocity

## Abstract

This investigation examined the association between on‐water rowing stroke‐rate (SR), velocity and heart‐rate (HR) in highly trained rowers (*n* = 8 male; *n* = 8 female and 19.3 ± 1.1 year) over a 4‐month real‐world (i.e., variable environment and boat class) training period. On‐water SR, prognostic velocity (percent of world's best velocity) and HR were captured for 1453 sessions via smart‐watch and chest‐strap HR monitor. Data was filtered and smoothed with individual HR training zones identified (T1–T5). Linear mixed modeling and an overlapping index ($\hat{\eta }$) were used to assess differences in SR and prognostic velocity between HR zones. Correlation coefficient (*r*) was used to assess the SR and prognostic velocity relationship and progression of prognostic velocity at a SR of 20spm over time. There were significant differences in SR and prognostic velocity between HR zones (T1>T2>T3>T4>T5, *p* < 0.001); however, data overlap between adjacent zones was substantial for both variables ($\text{SR}:\hat{\eta }$ = 0.69–0.85; prognostic velocity: $\hat{\eta }$ = 0.46–0.86). A significant, positive correlation (*r* = 0.50 and *p* < 0.001) between SR and prognostic velocity was found. Progression of prognostic velocity at SR 20spm over the study duration was trivial (*r* = −0.01 and *p* = 0.71). Variables commonly used to prescribe and describe on‐water rowing training showed large variability in a real‐world training environment impacting the ability to accurately monitor training performance and progression.

## Highlights

- Data from this study suggested that in a real‐world training environment, stroke rate and prognostic velocity show large spread when examined within heart rate training zones resulting in substantial data overlap between adjacent zones ($\hat{\eta }$ = 0.69–0.85).
- Using these variables in isolation to prescribe training may not result in the desired training stimulus, whilst performance monitoring using these variables may not accurately reflect athlete progression.
- Consideration should be given to the goal of on‐water rowing sessions (physiological and technical) as well as the context under which on‐water data is collected (e.g., flow, environmental conditions, session type and boat class) if using these variables for prescription and description purposes.

## 1 INTRODUCTION

Rowing athletes are regarded as some of the most physically talented in the world, possessing a combination of elite physiology and strength. Training for the 2000m race distance (five to eight min duration) places high metabolic demands on the athlete (Winkert et al., 2022‐). However, rowing performance also relies on the effective application of skill and technique in the on‐water environment, and therefore, accurate training prescription and performance monitoring methods on‐water are essential for holistic athlete development.

On‐water rowing often makes up >50% (10–12 h per week) (Tran et al., 2015; Treff et al., 2021) of overall training time in elite rowers and is commonly prescribed and described according to three variables—stroke‐rate (SR), velocity and heart‐rate (HR). Stroke‐rate is the simplest variable used to control rowing training intensity, and the relationship between SR and various rowing performance parameters has been previously researched (Held et al., 2020; Hofmijster et al., 2007; Kleshnev, 2001, 2010; Martin et al., 1980). Importantly, there is a well‐established relationship between SR and velocity in race scenarios (Martin et al., 1980; Holt et al., 2020‐; Holt et al., 2022). Generally, lower SRs translate to slower boat velocities and will elicit lower physiological stress (HR) due to the increased between stroke recovery time and reduced frequency of muscle contractions compared to higher SRs (Kleshnev, 2020, 2021a). As such, on‐water sessions are often prescribed at a low SR (<22spm), which enables large volumes of training to be completed without autonomic nervous system disturbance (Seiler et al., 2007). This allows for skill and technical reinforcement such that force per stroke and stroke length are developed alongside the development of the aerobic energy system, which are key indicators of performance (Kleshnev, 2020). As SR increases, the power and force requirements of the athlete are increased (Baudouin et al., 2002; Kleshnev, 2021a), which translates to a faster boat velocity (Kleshnev, 2001) and greater neuromuscular load (Kleshnev, 2020), in turn increasing the physiological intensity (HR) of effort. As such, on‐water training sessions may include higher SR (>24spm) intervals or stints of race‐intensity bursts (>30spm) which allow athletes to practice (and maintain) the biomechanics of a higher rate (Kleshnev, 2021b) as well as provide a high‐intensity training stimulus for athlete adaptation (Ní Chéilleachair et al., 2017).

Such relationships have also been scaled to inform training standards and monitor rowing performance over time (Rice, 2022) based on the idea that if an athlete can increase their velocity at a lower SR, race velocity will also improve (Kleshnev, 2020). A practical example of this exists in the Australian High‐Performance Rowing system, where the goal for underage athletes (<23 years) is to hit 80% prognostic velocity (percent of world's best velocity for specific boat class) at 20spm with a moderate aerobic intensity (HR) (Rice, 2022). Thus, monitoring prognostic velocity at a given SR over time should indicate on‐water rowing performance progression. One approach to this may be to target structured sessions where weather conditions are mild and athlete motivation is high, albeit these sessions are likely sporadic in occurrence. Alternatively, it may be assumed that large data capture over time will balance out confounding factors (environmental conditions and athlete performance fluctuation) such that prognostic velocity at set SR may be tracked.

In a real‐world training environment, however, there are numerous other factors that may influence SR, velocity and HR. The environment (i.e., wind direction and speed, water and ambient temperature and water flow) may impact boat velocity as well as an athletes' technique as they attempt to overcome varying conditions (Binnie et al., 2023; Kleshnev, 2009; Smith et al., 2011). Unfortunately, environmental conditions are complex to capture and account for, and there is currently no standardised method for collecting and reporting environmental data in rowing (Binnie et al., 2023). Additionally, it has been suggested in other on‐water racing sports (i.e., kayaking) that the ability of an athlete to modulate their intensity for any given SR may cause discrepancies between intensity measures (SR, HR and power output) (Hogan et al., 2020). Consequently, this self‐modulation of intensity at any given SR has the potential to impact the relationship between SR, velocity and HR. Thus, the interplay between SR, velocity and HR needs to be assessed in a real‐world training environment where such factors are at play as it may influence the resultant training stimulus and adaptive response. Previous literature documenting on‐water rowing training is limited to summary data or data captured in training diaries (Arne et al., 2009), as continuous data capture is complex and with significant resource cost. Recently, however, Watts and colleagues (Watts et al., 2022) suggested that a commercially available smart‐watch may provide a practical and reliable method of capturing on‐water rowing SR and velocity in conjunction with HR at scale. Using such technology, it becomes possible to gain an understanding of the interaction between these variables in the real world (e.g., variable environment, athlete effort level and crew combinations), which may have implications for the prescription and quantification of on‐water training.

Therefore, the aim of this investigation was to capture on‐water SR, prognostic velocity and HR over a 4‐month period from a group of highly trained under‐23 rowing athletes to explore the associations between these variables in a real‐world training setting. It was hypothesized that the relationship between variables during on‐water rowing training would be highly variable due to the impact of real‐world factors (i.e., environmental conditions, individual athlete technical ability and intensity self‐modulation).

## 2 MATERIALS AND METHODS

Sixteen highly trained (McKay et al., 2022) (*n* = 8 male, *n* = 8 female and age = 19.7 ± 1.02 years (M); 18.9 ± 0.98 years (F); body mass = 88.4 ± 7.87 kg (M); 70.9 ± 7.10 kg (F), V̇O_2peak_ = 5.42 ± 0.29 L.min^−1^ (M); 3.64 ± 0.22 L.min^−1^ (F), 12 state team representation and 4 national team representation) under‐23 rowing athletes were recruited to participate in this study. Ethics approval was granted by the Institutional Human Research Ethics committee (RA/4/20/5848). Written informed consent was obtained from each participant (parent/guardian for participants under 18 years) prior to undertaking the investigation.

For the determination of V̇O_2peak_, lactate thresholds 1 and 2 (LT1 and LT2) and training intensity zones, athletes completed a 7 × 4min graded exercise test on a rowing ergometer (Concept II, Morrisville, NC) as per the Rowing Australia Seven Step Rowing Protocol (Rice, 2016) prior to the commencement of training data collection. Average power was recorded for each step, and an earlobe capillary sample was assayed for lactate concentration (Arkay Group, Kyoto, Japa) in the 1‐min rest following completion of each step. Heart‐rate was captured continuously throughout the test (Wahoo Fitness, Atlanta, USA), and expired gas was analyzed for O_2_ and CO_2_ concentrations (ParvoMedics, Salt Lake City, UT) with V̇O_2peak_ taken as the highest value obtained over a full minute. The relationship between power output and blood lactate during the test was used to determine LT1 and LT2 for each athlete using the modified *D*_max_ method (ADAPT Software, Canberra, Australia). A five‐zone training intensity model was determined using LT1 and LT2 as physiological landmarks for the associated HR and by anchoring the lowest and highest intensities to the HR corresponding to 50% and 100% V̇O_2peak,_ respectively. Descriptors for HR training zones were as follows: T1, the intensity between 50% V̇O_2peak_ and the midway point between of 50% V̇O_2peak_ and LT1; T2, the intensity between the top of T1 and LT1; T3, the intensity between LT1 and 95% of LT2; T4, the intensity between 95% and 102% of LT2 and T5, the intensity between 102% of LT2 and 100% V̇O_2peak_ (Seiler et al., 2009; Watts et al., 2023).

Over a 4‐month period, SR, velocity and HR were captured for all on‐water rowing sessions using a 1 Hz smart‐watch (Garmin Forerunner 735XT, Garmin Ltd, USA) which was paired with a chest‐trap HR monitor via Bluetooth (Wahoo Fitness) for the measurement of HR. The smart‐watch has previously been deemed reliable for SR and velocity measurement during on‐water rowing by Watts and colleagues (Watts et al., 2022). Athletes wore the smart‐watch on their preferred wrist, used the ‘rowing’ activity profile to record, and had the data recording function set to ‘Every Second’, as per the recommendations of Watts and colleagues (Watts et al., 2022). Captured sessions were uploaded and stored on an online platform (TrainingPeaks, Louisville, USA) during the 4‐month period. Boat class was recorded for each athlete's on‐water sessions via a weekly training diary.

Data processing was conducted using RStudio (R: A language and environment, 2020). Filtering and smoothing processes described by Watts et al. (2022) were applied to the data: SR was filtered between 14 and 50spm; velocity was smoothed over a 15s moving average and filtered between 2.1 and 7.0 m.s^−1^. This process was aimed at eliminating extreme data points which result from performing technical warm‐up drills or turning the boat around. Velocity was converted to a prognostic velocity (percentage of senior world's best velocity for the respective boat class). Heart‐rate was classified according to the five‐zone training intensity distribution model (T1–T5). Data which was not classified into one of these zones (i.e., <T1) was filtered out of the database. Additional filters were applied to remove known sources of variation (such as instances of sharp accelerations and decelerations in boat velocity and the HR lag effect) through trial and error. A gradient filter was used to eliminate data points where one or more variables was changing rapidly (e.g., such as in a stationary race start). This resultant filter removed data points where SR changed by ≥ 1.5spm compared to the data point 5s prior and velocity changed ≥0.2 m.s^−1^ compared to the data point 3s prior. Additionally, a filter was applied to HR data such that if there were less than 15 consecutive points in a given HR zone (T1‐T5), that data was removed (to account for brief periods in a lower zone as athletes commenced a higher‐intensity effort).

Summary data was created by averaging SR and prognostic velocity for HR zones T1–T5 within each session for each athlete. Heart‐rate was chosen as the main grouping variable for analysis as there is literature to support the use of HR training zones (Seiler et al., 2009), whereas SR and velocity zones are less established in rowing. Two additional summaries were produced to assess the SR and velocity relationship. For this analysis, data was filtered to include only sessions completed in the single scull (1X). One dataset was produced by grouping SR into zones: 14–18spm, 19–21spm, 22–24spm, 25–27spm, 28–30spm, 31–33spm, 34–36spm, 37–39spm, 40–42spm, 43–45spm, 46–50spm and calculating the average SR and prognostic velocity within each zone for each session and athlete. To assess the progression of prognostic velocity for a given SR throughout the 4‐month of training, a subset of this data was generated by excluding all SRs except for those between 19 and 21spm. For this dataset, data was grouped by athlete and by session and average prognostic velocity was calculated.

Statistical analysis was completed using RStudio (R: A language and environment, 2020), and data are presented as mean ± standard deviation (SD). The distribution of SR and prognostic velocity for each HR zone was examined and presented via boxplots. Linear mixed modeling using the R package ‘lmerTest’ was used to analyze the data with athlete‐ID included as a random intercept. All modeling was estimated using Restricted Maximum Likelihood. *p‐*values were obtained using Satterthwaite's method with Kenward‐Rodger degrees of freedom. Post‐hoc analysis was conducted using a false discovery rate correction factor from the R package ‘emmeans’ with upper and lower 95% confidence limits (CLs) also determined. An overlapping index ($\hat{\eta }$) was calculated using the R package ‘overlapping’ to determine the amount of data shared between HR training zones for both SR and prognostic velocity, where $\hat{\eta }$ = 0 indicates distributions are completely separated and $\hat{\eta }$ = 1 indicates distributions are exactly the same (Pastore et al., 2019). The relationship between average SR and average prognostic velocity and the progression of prognostic velocity at a SR of 20spm (19–21spm) over time were examined via Pearson's product‐moment correlation (*r*). The magnitude of correlation was assessed with the following thresholds: *r* < 0.1, trivial; *r* = 0.1–0.3, small; *r* = 0.3–0.5, moderate; *r* = 0.5–0.7, large; *r* = 0.7–0.9, very large; *r* = 0.9, nearly perfect and *r* = 1, perfect. (Hopkins) Statistical significance was accepted at *a*<0.05.

## 3 RESULTS

Following all data filtering and processing, 68.9% of total captured data remained. A total of 1453 training sessions were captured and included in the final analysis (*n* = 90 ± 13 sessions per athlete and duration = 40.7 ± 21.2 min per session). Boat class session count was as follows: single scull (1X) = 601, coxless pair (2‐) = 489, double scull (2X) = 78, coxless four (4‐) = 87, quadruple scull (4X) = 35 and coxed eight (8+) = 163.

**FIGURE 1:**
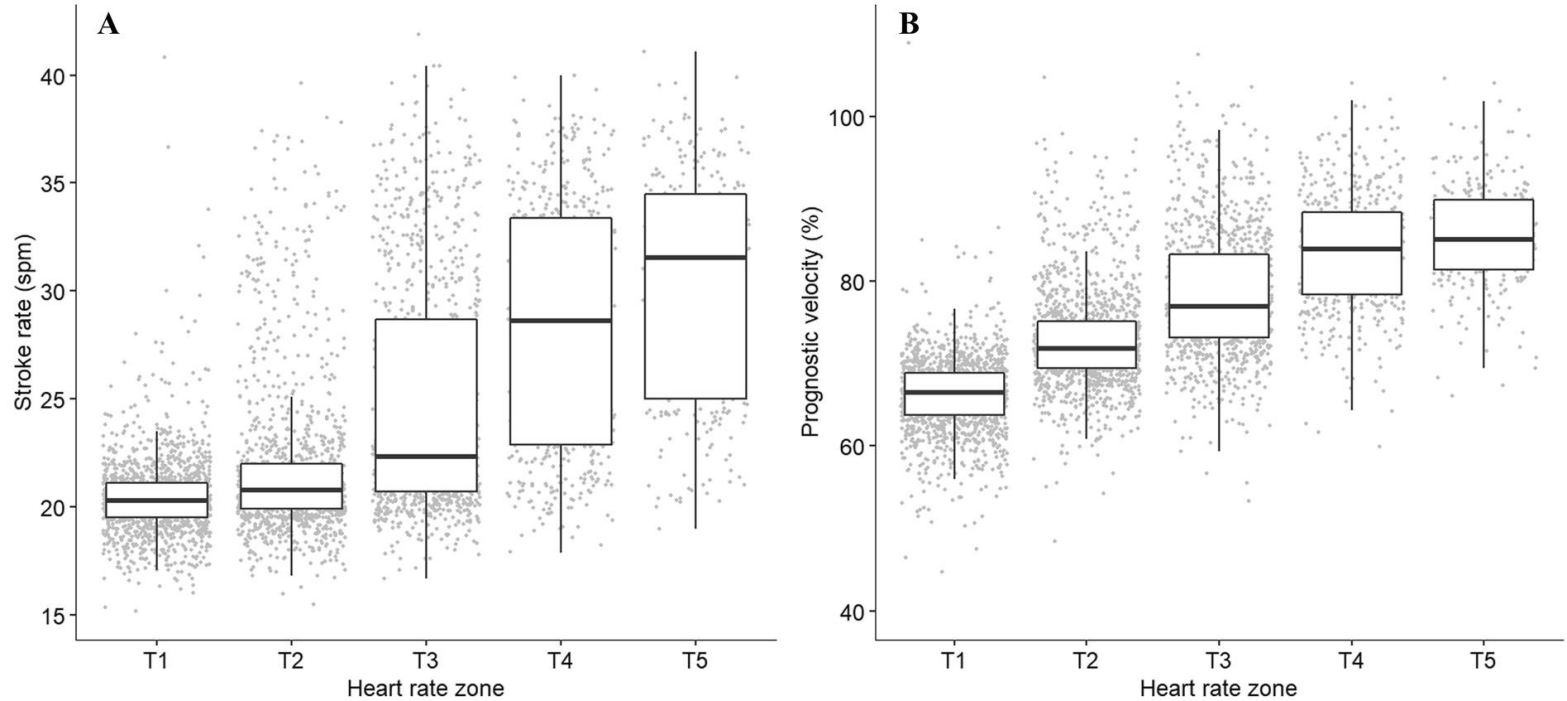
Distribution of stroke rate (A) and prognostic velocity (B) according to the five heart rate training zones (T1‐T5). Box represents median ± interquartile range (IQR) with whiskers representing quarter 1–1.5*IQR and quarter 3 + 1.5*IQR. T1, the intensity between 50% V̇O_2peak_ and the midway point between of 50% V̇O_2peak_ and the first lactate threshold (LT1); T2, the intensity between the top of T1 and LT1; T3, the intensity between LT1 and 95% of the second lactate threshold; T4, the intensity between 95% and 102% LT2 and T5, the intensity between 102% of LT2 and 100% V̇O_2peak._

The distribution (median ± interquartile range) of SR and prognostic velocity according to HR training zones is presented in Figures 1A,B, respectively. The average SR and prognostic velocity for each HR training zone with 95% confidence limits and overlapping index ($\hat{\eta }$) are presented in Table 1. Results from linear mixed modeling and post‐hoc analysis indicated that SR and prognostic velocity increased with HR training intensity zone (Table 1, T1>T2>T3>T4>T5, all *p* < 0.001).

**TABLE 1:** Mean ± standard deviation (SD) for stroke rate (SR) and prognostic velocity for each training zone (T1‐T5) with upper and lower 95% confidence limits (CLs), difference (diff) between zone means and overlapping index ($\hat{\eta }$).

Heart rate zone	Mean ± SD	95% CL	T2 diff ($\hat{\eta }$ )	T3 diff ($\hat{\eta }$)	T4 diff ($\hat{\eta }$)	T5 diff ($\hat{\eta }$)
Stroke rate (spm)						
T1	20.4 ± 1.87	20.0–20.9	−1.35 (0.85)	−4.35 (0.56)	−7.99 (0.30)	−10.21 (0.18)
T2	21.8 ± 3.55^a^	21.3–22.3	‐	−3.00 (0.69)	−6.64 (0.43)	−8.85 (0.30)
T3	24.8 ± 5.49^a^ ^,^ ^b^	24.3–25.3	‐	‐	−3.64 (0.70)	−5.85 (0.55)
T4	28.4 ± 5.72^a^ ^,^ ^b^ ^,^ ^c^	27.9–29.0	‐	‐	‐	−2.22 (0.85)
T5	30.6 ± 5.29^a^ ^,^ ^b^ ^,^ ^c^ ^,^ ^d^	30.0–31.3	‐	‐	‐	‐
Prognostic velocity (%)						
T1	66.2 ± 4.59	65.1–67.2	−6.77 (0.46)	−12.44 (0.26)	−17.9 (0.11)	−20.14 (0.07)
T2	72.9 ± 6.12^a^	71.9–73.9	‐	−5.67 (0.63)	−11.13 (0.36)	−13.37 (0.26)
T3	78.6 ± 7.91^a^ ^,^ ^b^	77.6–79.6	‐	‐	−5.46 (0.68)	−7.70 (0.55)
T4	84.1 ± 7.14^a^ ^,^ ^b^ ^,^ ^c^	83.0–85.2	‐	‐	‐	−2.25 (0.86)
T5	86.3 ± 6.31^a^ ^,^ ^b^ ^,^ ^c^ ^,^ ^d^	85.1–87.5	‐	‐	‐	‐

^a^ Significantly different from T1.

^b^ Significantly different from T2.

^c^ Significantly different from T3.

^d^ Significantly different from T4.

**FIGURE 2:**
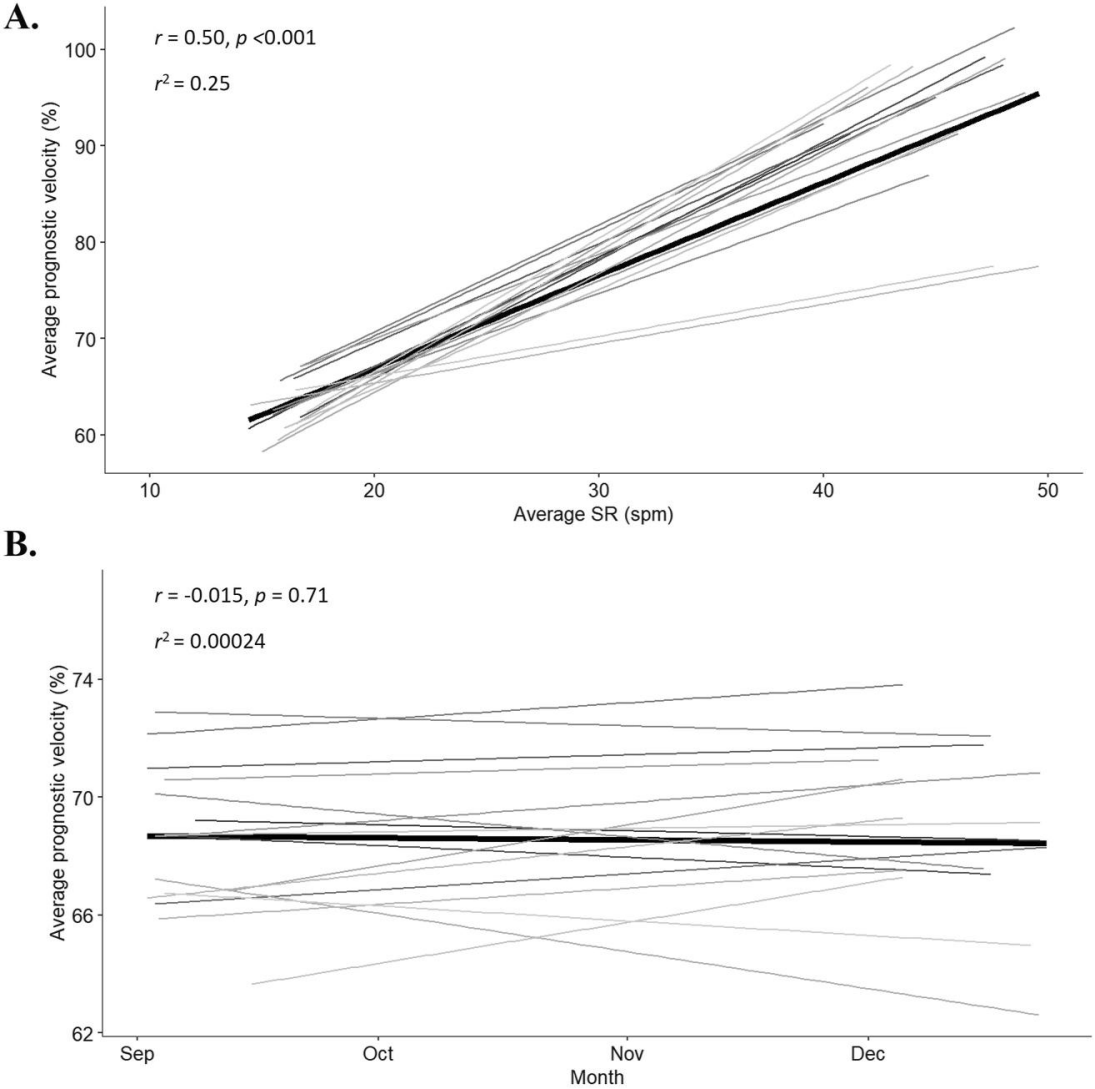
Overall regression lines are depicted in bold and individual athlete regression lines in grey for: A) sessional average stroke rate (SR, spm) and prognostic velocity (%) in single scull (1X) boats and B) prognostic velocity at a SR of 20 spm (19–21 spm) in 1X boats for the duration of the study (September–December). *Stroke rate was calculated as the average of the following zones: 14–18spm, 19–21spm, 22–24spm, 25–27spm, 28–30spm, 31–33spm, 34–36spm, 37–39spm, 40–42spm, 43–45spm and 46–50spm.

The relationship between average SR and average prognostic velocity in 1X boat class is presented in Figure 2A. There was a moderate, positive correlation between SR and prognostic velocity which was statistically significant (*r* = 0.50 and *p* < 0.01) with individual athlete correlations ranging from small (*r* = 0.20) to very large (*r* = 0.70). The progression of prognostic velocity at a SR of 20spm (19–21spm) over time in 1X boat class is presented in Figure 2B. There was a trivial, negative relationship over time which did not reach significance (*r* = ‐0.015, *p* = 0.71) and individual athlete correlations ranged from small negative (*r* = −0.28) to moderate positive (*r* = 0.41).

## 4 DISCUSSION

This investigation explored the association between on‐water rowing SR, velocity and HR over a 4‐month period from 16 highly trained rowing athletes. Despite significant differences in SR and prognostic velocity between HR training zones, there was large variability and spread of SR and prognostic velocity within each zone resulting in substantial data overlap (Figures 1A andB, Table 1). Additionally, the strength of the relationship between SR and prognostic velocity varied considerably between athletes with no apparent trend for changes in prognostic velocity at a set SR over time. This may have practical implications for use of these common rowing variables when considering an athlete's on‐water training prescription and performance progression.

The data captured in this investigation indicated that SR and prognostic velocity increased with intensity (HR), which supports the idea that higher SRs and faster velocities require greater force application from the athlete, and thus, increases in the intensity (HR) of effort. However, SR and prognostic velocity showed large variability within each HR training zone and a high degree of data overlap between adjacent HR training zones (i.e., T1 and T2; T2 and T3; T3 and T4 and T4 and T5), making it difficult to decipher between zones. This was further compounded by the fact adjacent HR training zones only differed between 1.3 and 3.6spm for SR and 2.2%–6.7% (0.1–0.4 m.s^−1^) for prognostic velocity (Table 1). Often, in training, SR will be prescribed as a range rather than a single number (e.g., SR of 18–20spm) (Rice, 2022). As such, the bandwidth of SR prescription (2spm) may be equal to or greater than the average difference between adjacent physiological training zones. Additionally, it should be considered that our dataset contained over 1400 sessions, whereas coaches and practitioners are often examining a much smaller dataset from a single session or athlete. As such, with a much smaller dataset, our confidence in distinguishing between zones diminishes if using these variables in isolation to prescribe or describe training.

The importance of being able to clearly decipher between training zones was evident in a study by Watts and colleagues (Watts et al., 2023). This investigation suggested highly demarcated physiological training zones were key to understanding training adaptation and performance enhancement in rowing, with time in T2 HR zone important for aerobic (V̇O_2peak_) development and time in T4 HR zone important for LT2 power and 2000m ergometer performance. Thus, coaches may consider prescribing training by HR to ensure the desired physiological stimulus is being achieved, albeit this may be at the cost of specific skill development and the intent of the rowing stroke, which are also key determinants of success. Alternatively, if SR is solely used as the prescription variable, technique and development of force per stroke may benefit to the detriment of the specificity of the physiological training stimulus. It should also be acknowledged that the agreement between ergometer‐derived training zones and on‐water training can be somewhat variable between athletes (Vogler et al., 2010), albeit this method of training zone determination has been successfully used to quantify rowing training in applied research (Watts et al., 2023). Consequently, due to the noise associated with SR, velocity and HR during on‐water rowing, the desired training stimulus and resultant training adaptation for athletes may be impacted if these variables are being used in isolation to prescribe training.

The high variability of data may also impact the practicality of using these variables for performance monitoring. If we consider that SR and velocity are positively related in race scenarios (Martin et al., 1980; Holt et al., 2020‐; Holt et al., 2022), monitoring prognostic velocity at a given SR over time appears a logical method of tracking athlete performance progression. The current investigation suggested that SR and prognostic velocity demonstrate a moderate positive correlation in a training environment, albeit there was large individual variation (Figure 2A) which may somewhat be attributed to differences in athlete skill, as well as external (environmental) factors. Additionally, prognostic velocity at a SR of 20spm is considered a key indicator of performance and progression in the Australian High‐Performance Rowing system. Prognostic velocity at a SR of 20spm (19–21spm) was examined over time in the current investigation, albeit no relationship was evident and this was consistent on an individual athlete level (Figure 2B). We may consider that the investigation length (4‐months) was not sufficient for changes in prognostic velocity to be evident. Although, given the variability of SR and prognostic velocity in HR zones discussed previously, it is likely that the lack of change in prognostic velocity over time is due to ‘noise’ associated with training in a real‐world environment. Limiting such analysis to more controlled training sessions (capped rating, maximum athlete intent and same stretch of water) may be an approach to overcome such high data variability; however, this method may also be flawed when we consider that reducing session sample size could inflate the impact of other external variables (environment).

Environmental conditions are perhaps the most obvious source of data variability in the current investigation. Over the 4‐month training period, athletes were exposed to a range of environmental conditions, although details on these conditions were not captured due to the complexity of continuous measurement and lack of standardised procedure (Binnie et al., 2023). Boat velocity may be the main variable impacted by the environment of which there are numerous aspects to consider, such as wind (direction and speed), temperature (ambient and water) and water flow (Hogan et al., 2022; Kleshnev, 2009, 2020). Other factors to consider which may impact the interaction between a boat and the environment include athlete technique (squaring of blade), athlete size (male vs. female; lightweight vs. heavyweight) and boat size (1X vs. 8+) (Kleshnev, 2009). Additionally, an athlete's technique (SR) and physiological response (HR) may also be influenced by the environment in attempt to overcome variable conditions. Notably, both cool (10°C) and hot (35°C) ambient temperatures have been shown to elevate HR response during submaximal exercise compared to moderate temperatures (22°C) (No et al., 2016). Consequently, the conditions in which athletes trained on any given day had the potential to substantially influence SR, prognostic velocity and/or HR even if session prescription remained consistent. Although the environment may be a major confounding factor contributing to data variability, this is reflective of what athletes typically encounter during real‐world training, adding to the problem of using these variables in isolation for training prescription and description purposes.

Other sources of data variability may be attributed to the rowing system and the nature of training. Unlike other endurance sports, such as cycling, rowing is not a geared system and athletes have the ability to modulate their intensity at any given SR. Discrepancies between intensity measures (SR, HR and power output) have been found in other on‐water racing sports due to this self‐modulation (Hogan et al., 2020). As such, the ability of athletes to self‐modulate intensity may have resulted in discrepancies between variables in the current investigation further adding to data variability. Session prescription may be another factor contributing to data variability such that a steady‐state rowing session of 20km with a SR of 20spm is likely to elicit a different HR response compared to performing 1500m intervals at a SR of 20spm with ‘full pressure’ applied, even though the SR is the same. Importantly, it has been determined that both HR and SR misrepresented supramaximal sprint training in another water‐based paddling sport (kayak). In the sport of kayaking, Hogan and colleagues (Hogan et al., 2021) found that quantification of supramaximal interval training using HR underestimated training intensity relative to power, while quantification using SR was limited due to fatigue associated with high‐intensity training. Additionally, athlete or crew technical ability and degree of technical efficiency may impact physiological (HR) response and velocity such that an increase in SR and force application may result in an increase in HR but not boat velocity (Buckeridge et al., 2015). Consequently, such discrepancies add further limitation to the use of SR, prognostic velocity and HR in isolation as either a training prescription or performance monitoring tool.

While SR, velocity and HR remain key indicators of rowing performance and internal response to training, their use in a real‐world training setting is flawed. One potential avenue which may improve our understanding of the interplay between these variables is through the addition of on‐water power output. The relationship between power and velocity in water‐based sports is theoretically curvilinear (Hill et al., 2009; Hogan et al., 2022; Holt et al., 2022), although it may be impacted by environmental conditions and athlete characteristics (e.g., size and technical efficiency). Despite this, the literature suggests that power may offer a more direct assessment of exercise intensity and boat performance (Holt et al., 2020‐; Hogan et al., 2022; Lintmeijer et al., 2019). Holt and colleagues (Holt et al., 2022) found that when accounting for measures of SR and head wind, power output during a 2000m rowing race had the largest modifying effect on predicted boat velocity. Importantly, sprint kayaking is another water‐based paddling sport which encounters some of the same training prescription and description issues as rowing. Work by Hogan and colleagues (Hogan et al., 2020) in this area examined the training response of SR and HR compared to power output and showed that SR and HR misrepresented the time spent in training zones when intensity was controlled by power output. A follow‐up study by Hogan and colleagues (Hogan et al., 2022) showed power could more accurately account for the influence of water flow as compared to global positioning system derived velocity, making it a more appropriate measure for quantifying external training load on flowing waterways. Additionally, on‐water power output may be useful as an instantaneous feedback tool with one investigation reporting a 65% improvement in rowing training intensity adherence when using power compared to coach feedback, boat velocity and SR alone (Lintmeijer et al., 2019). These studies provide some evidence to support the use of power in water based sports to obtain more objective measures of intensity in real‐world conditions. However, it should be highlighted that there may be limitations in using laboratory (ergometer) derived power measures for on‐water power prescription due to differences in the measured power between the two devices (ergometer vs. on‐water instrumentation) (Holt et al., 2021; Kleshnev, 2005). Additionally, measured power output on‐water may vary depending on the type and location of the rowing instrumentation (e.g., oarlock vs. oar shaft) (Holt et al., 2021). Ultimately, to improve upon the current training prescription and description practices in on‐water rowing, further research should consider power measurement alongside other training metrics, such as SR, velocity and HR, across variable environmental conditions and boat classes.

Of course, there are some limitations of this investigation. Firstly, the device used to monitor SR and velocity (Garmin Forerunner 735XT smart‐watch) has a degree of error and uses proprietary algorithms for the calculation of variables, which may have inflated the variability of data collected. Additionally, athletes were in a mix of sweep (one oar) and scull (two oars) boats with different crew combinations. Although this is reflective of real‐world practices, it may have had technical influence which resulted in variation to SR, prognostic velocity and/or HR. Finally, environmental conditions (i.e., water flow, water temperature, wind speed and direction and ambient temperature) which may have given some additional context to the data were not recorded due to the complexity of continuous data capture from multiple locations over an extended period.

In summary, this investigation demonstrated that the variables used to prescribe and describe on‐water rowing training (SR, prognostic velocity and HR) shows large variability in a real‐world training environment. Consequently, training prescribed according to an assumed relationship between SR, velocity and HR may not elicit the desired training stimulus potentially impacting athlete adaptation to training. Additionally, monitoring athlete performance through changes in prognostic velocity at a set SR may not accurately reflect rowing progression. As such, practitioners should be clear on the session goals (physiological and technical) if using these variables to prescribe on‐water training, whilst consideration should be given to the context in which on‐water rowing data is collected (e.g., flow, environmental conditions, session type and boat class). The addition of power output measured alongside SR, velocity and HR should be considered to further improve on‐water rowing training prescription and description practices.

## CONFLICT OF INTEREST STATEMENT

The authors report no conflicts of interest.

## Data Availability

The datasets generated and/or analyzed during the current study are not publicly available due to the sensitive nature of athlete data in a sports institute setting, but are available from the corresponding author on reasonable request.
